# Supplementary figures and images for: A telomerase with novel non-canonical roles: TERT controls cellular aggregation and tissue size in Dictyostelium
Source: PLoS Genet. 2019 Jun 25;15(6):e1008188. doi: 10.1371/journal.pgen.1008188 (PMC6592521; doi:10.1371/journal.pgen.1008188)

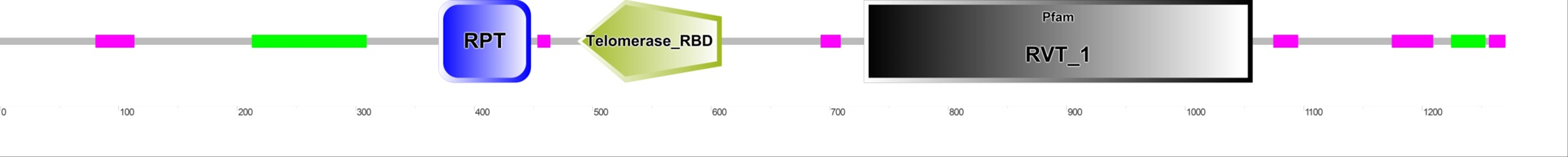

Supplement: S1 Fig — TERT protein contains the following domains: a reverse transcriptase (RVT) and an RNA binding domain (Telomerase_RBD). (TIF) [file pgen.1008188.s001.tif]

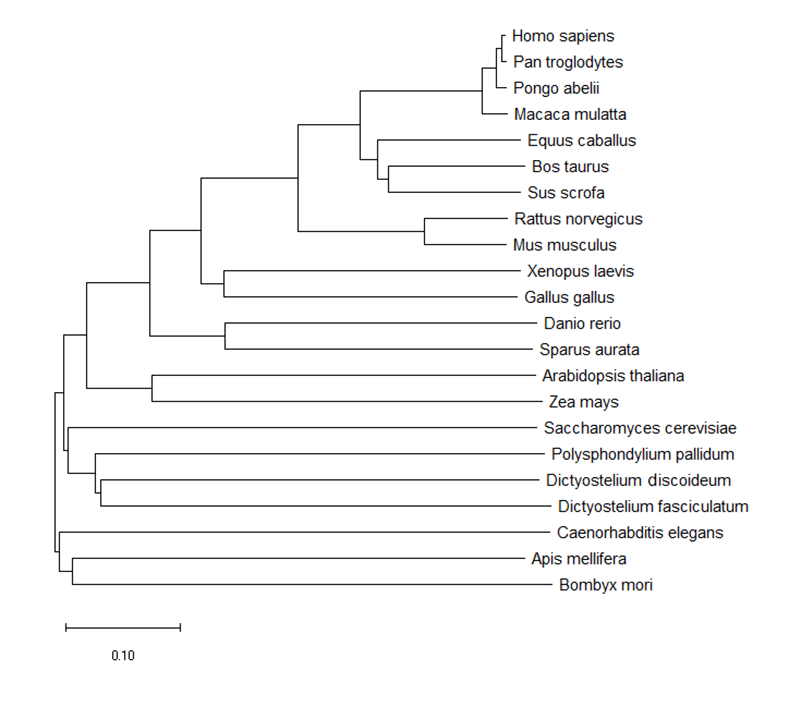

Supplement: S2 Fig — Neighbour-Joining Tree was constructed using Muscle alignment of MEGAX (Molecular Evolutionary Genetic Analysis X). (TIF) [file pgen.1008188.s002.tif]

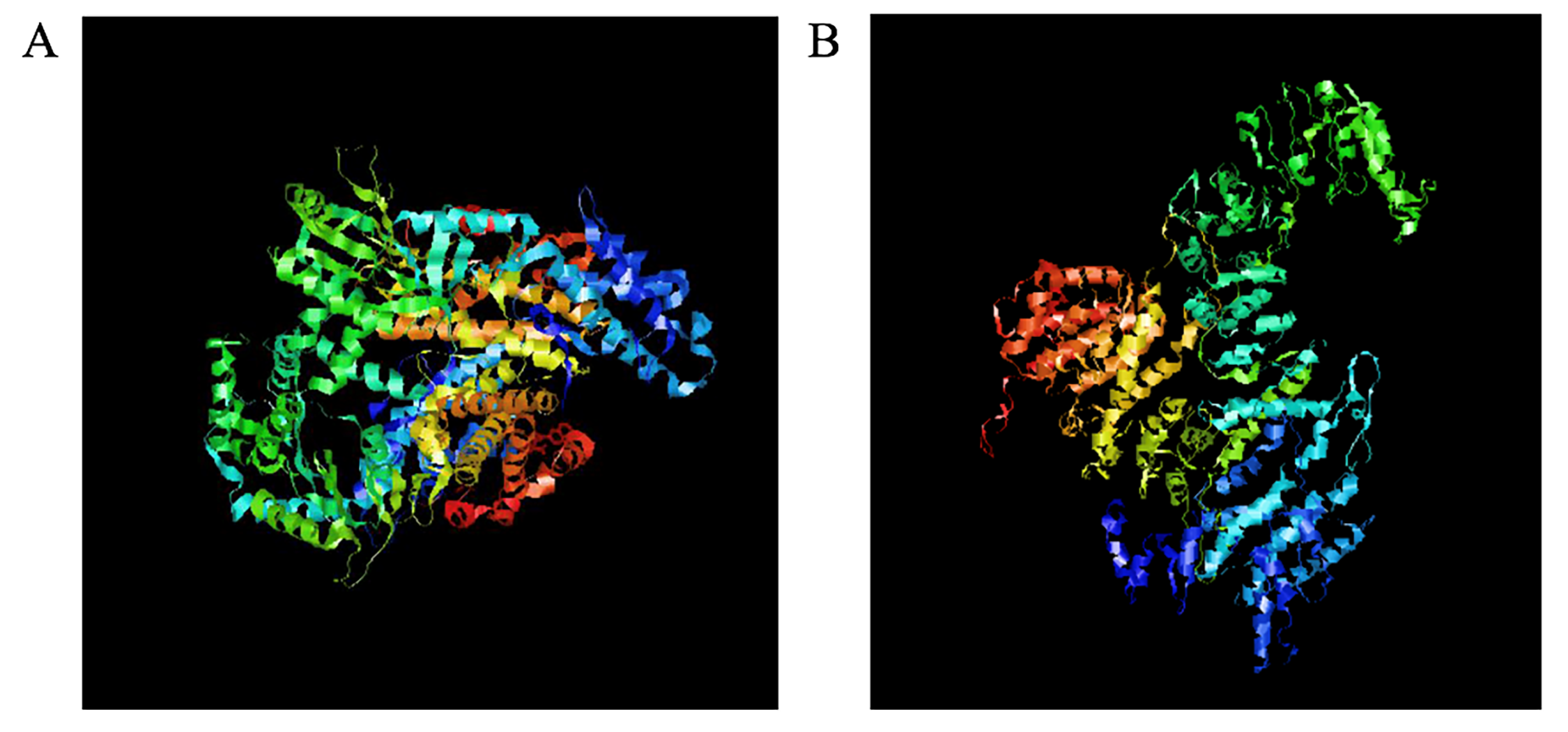

Supplement: S3 Fig — (A) Tribolium castaneum TERT. (B) D. discoideum TERT. The TERTs of Tribolium castaneum (which was used as a template for prediction) and D. discoideum have many structural similarities. (TIF) [file pgen.1008188.s003.tif]

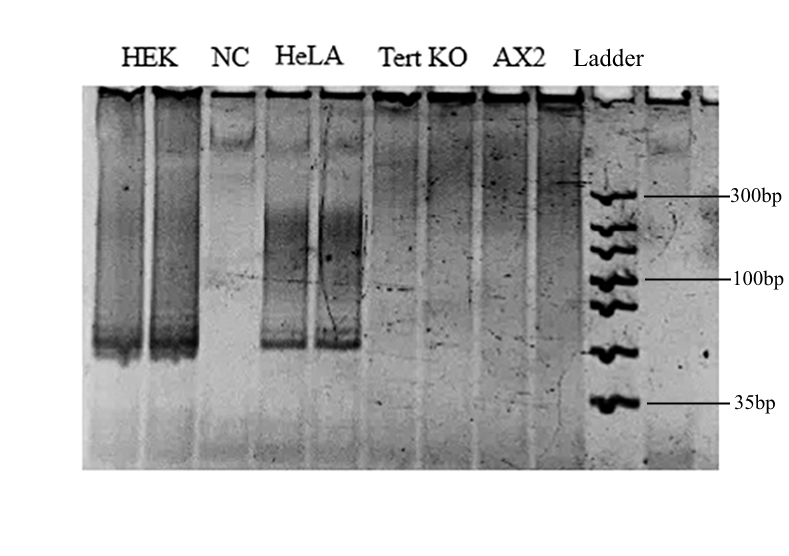

Supplement: S4 Fig — TRAP assay were performed for AX2 and tert KO. Human cell lines HEK and HeLa were used as positive controls. NC is an abbreviation for ‘No-Template’ control. TrackIT Ultra low range DNA ladder was used. (TIF) [file pgen.1008188.s004.tif]

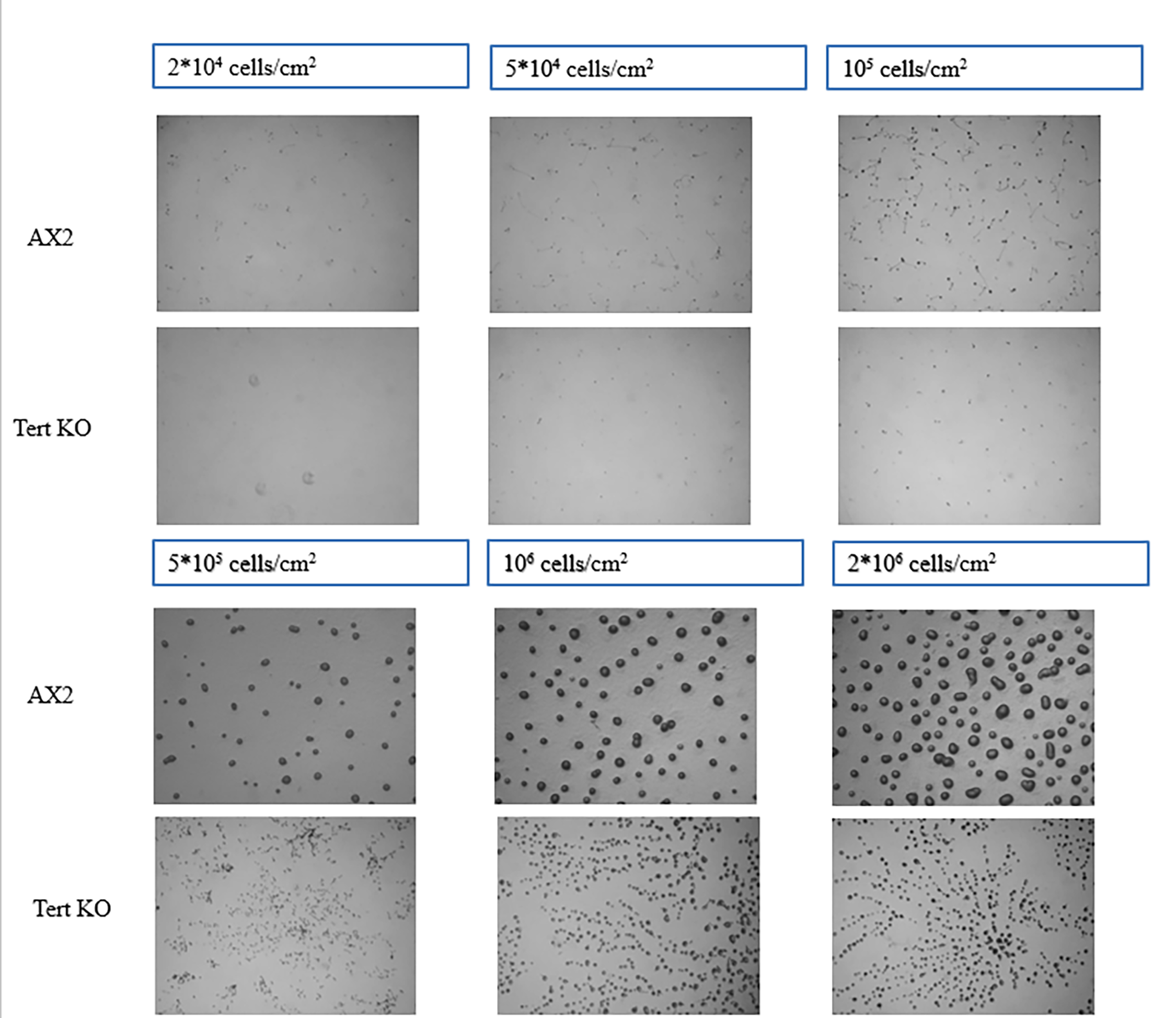

Supplement: S5 Fig — Development assay at different cell density (2x10^4^ cells/cm2 to 2x10^6^ cells/cm2). AX2 cells aggregate even at a cell density below 2x10^4^ cells/cm2, but tert KO fails to aggregate at such a density. Tert KO phenotype was not rescued even at higher cell density (2x10^6^ cells/cm2). (TIF) [file pgen.1008188.s005.tif]

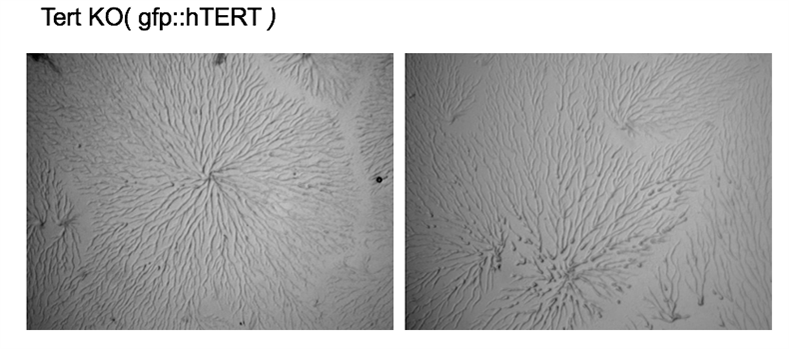

Supplement: S6 Fig — (TIF) [file pgen.1008188.s006.tif]

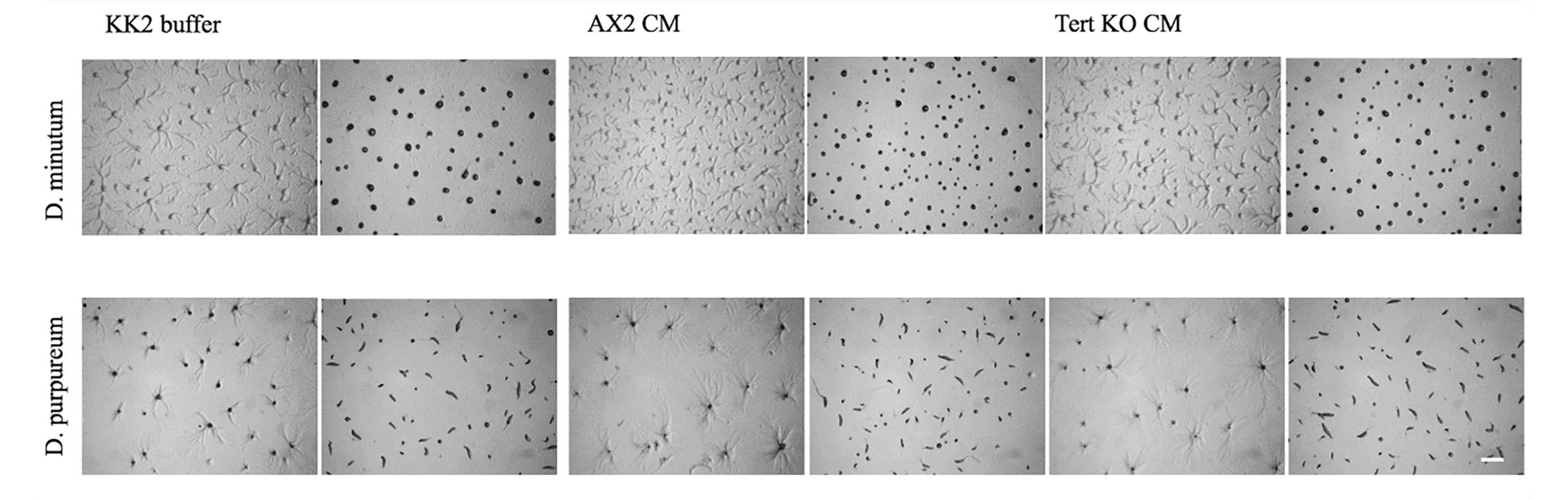

Supplement: S7 Fig — tert KO-CM did not alter the group size of other dictyostelids. Scale bar: 0.5 mm; (n = 3). (TIF) [file pgen.1008188.s007.tif]

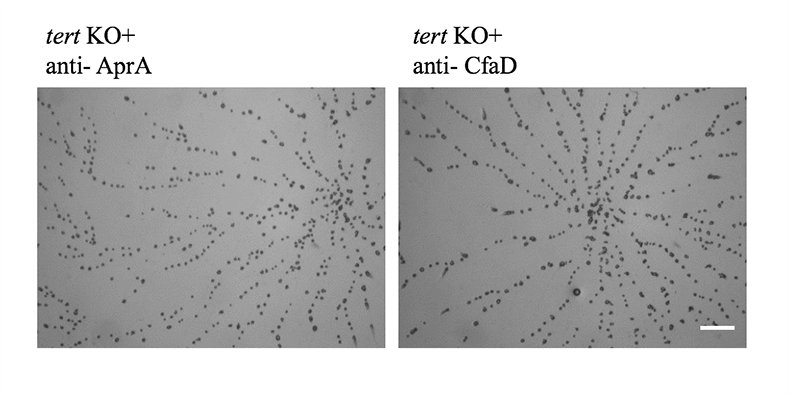

Supplement: S8 Fig — Scale bar: 0.5 mm; (n = 3). (TIF) [file pgen.1008188.s008.tif]

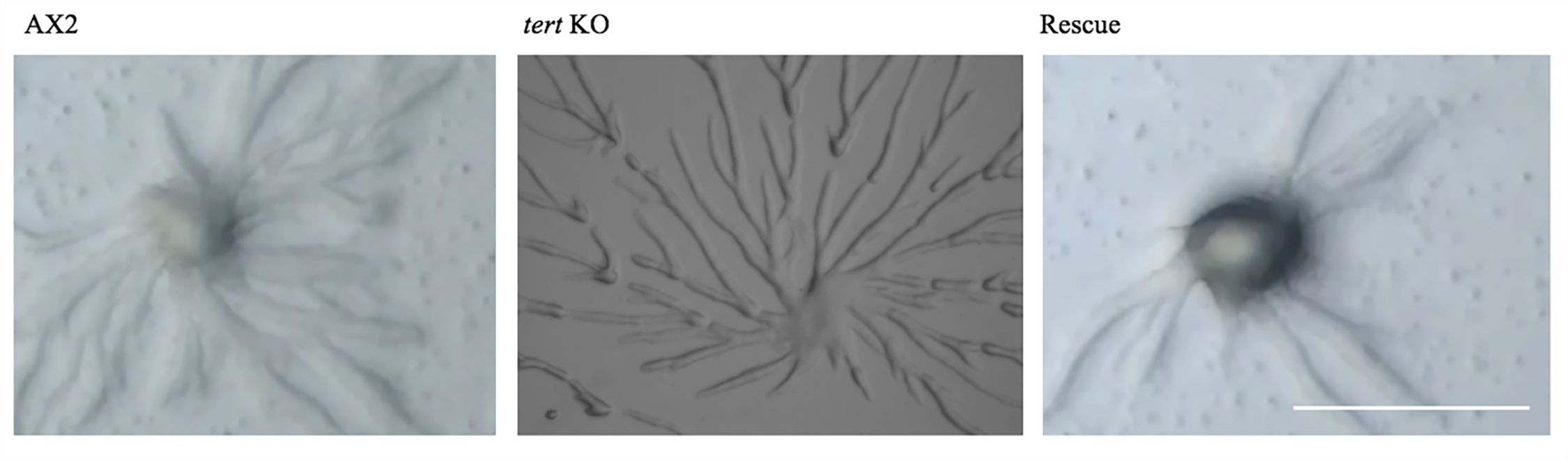

Supplement: S9 Fig — (TIF) [file pgen.1008188.s009.tif]

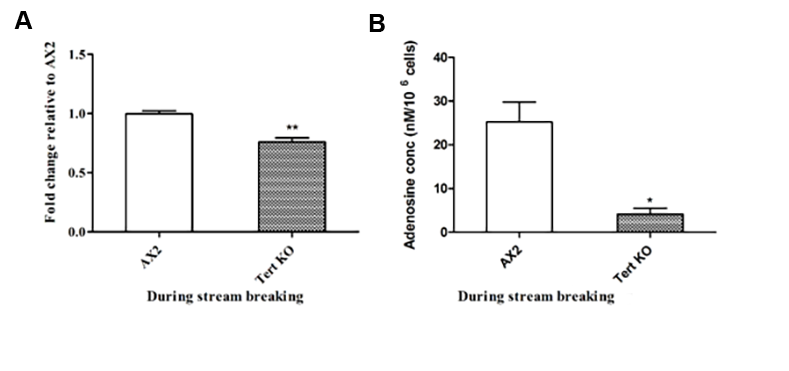

Supplement: S10 Fig — A) qRT-PCR of 5’NT during stream breaking. Fold change in mRNA expression is relative to AX2 at the indicated time points. rnlA is used as mRNA amplification control. B) Quantification of adenosine levels during stream breaking. Level of significance is indicated as *p<0.05, **p<0.01, ***p<0.001, and ****p<0.0001. (TIF) [file pgen.1008188.s010.tif]

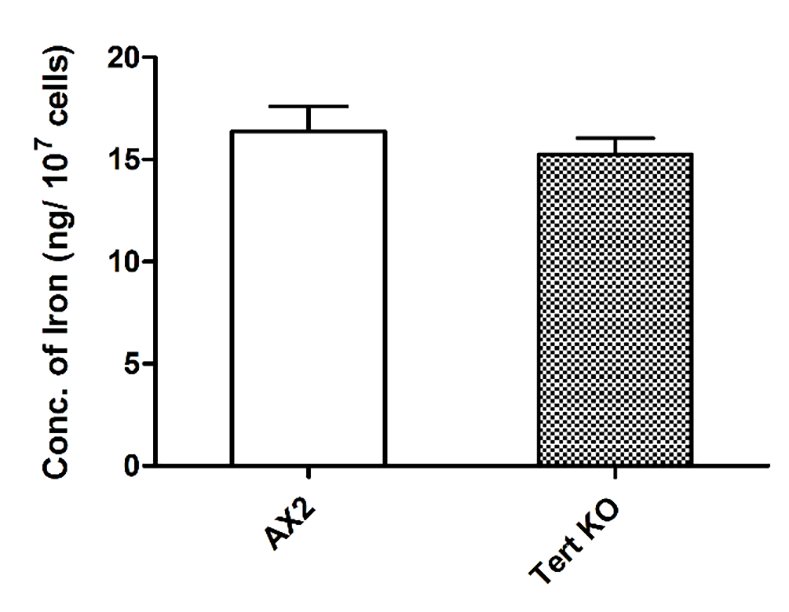

Supplement: S11 Fig — Iron levels were quantified by ICP-MS. Level of significance is indicated as *p<0.05, **p<0.01, ***p<0.001, and ****p<0.0001. (TIF) [file pgen.1008188.s011.tif]

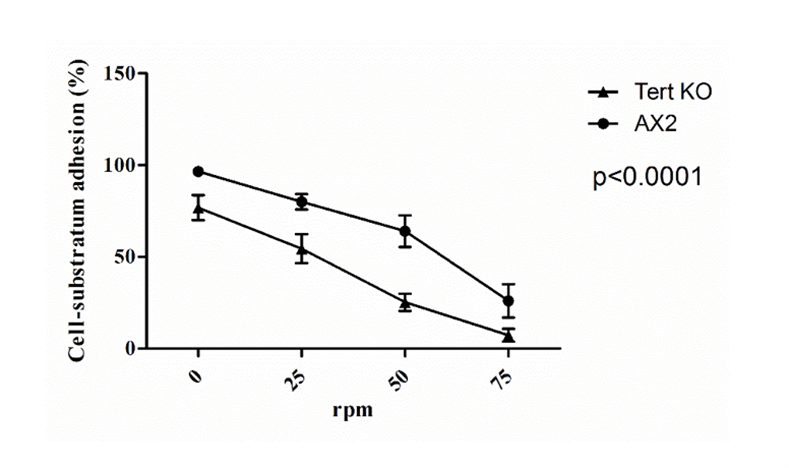

Supplement: S12 Fig — Cells were plated at a density of 1x10^5^ cells/ml, grown overnight, in an orbital shaker. Floating and attached cells were counted and percentage adhesion was plotted versus rotation speed; (n = 3). Both AX2 and tert KO exhibited a sheer force-dependent decrease in substratum adhesion and tert KO exhibited significantly reduced adhesion compared to AX2 cells. (TIF) [file pgen.1008188.s012.tif]

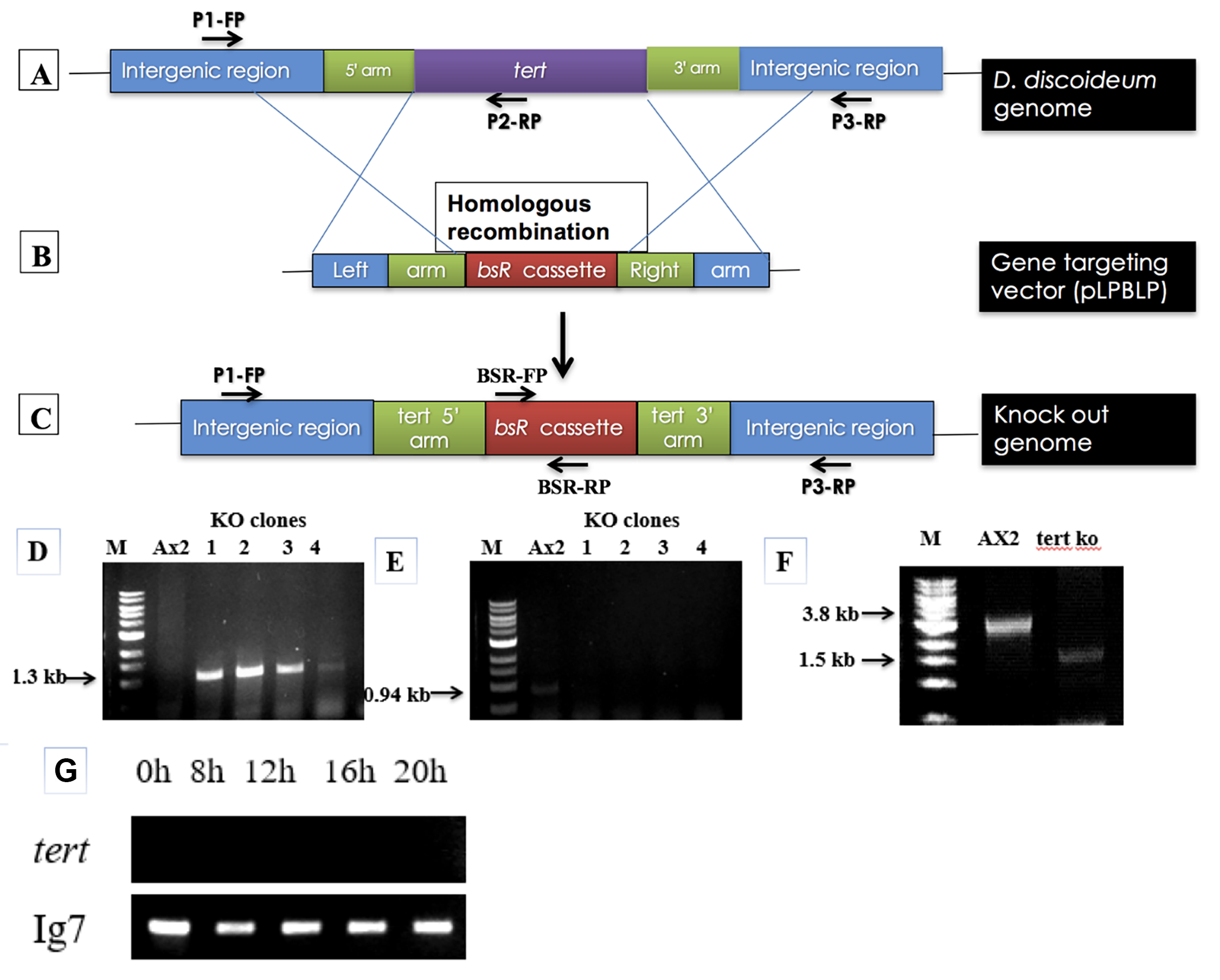

Supplement: S13 Fig — A) Physical map of tert gene in the genome. PCR primers are shown at positions where they bind. B) The targeting vector (pLPBLP) with sites of recombination and Blasticidin S resistance gene (Bsr). C) Physical map of the genome after targeted gene disruption. D) PCR amplification of DNA using primers that prime outside the vector (P1 FP) and inside the Bsr cassette (BSR RP); no amplicons were obtained from AX2. E) Amplification of the sequence immediately upstream of the tert gene (P1 FP) and within the tert gene (P2 RP), DNA amplification was observed only in AX2 and not in the tert KO clones. F) PCR of genomic sequences flanking the insertion site. A 3.8 kb fragment from AX2 and 1.5 kb amplicon from the tert KO were observed. G) RT-PCR of tert in the tert KO clone. Ig7 (rnlA) was used as an mRNA amplification control. (TIF) [file pgen.1008188.s013.tif]
