# Supplementary material for: A telomerase with novel non-canonical roles: TERT controls cellular aggregation and tissue size in Dictyostelium
Source: PLoS Genet. 2019 Jun 25;15(6):e1008188. doi: 10.1371/journal.pgen.1008188 (PMC6592521; doi:10.1371/journal.pgen.1008188)
Supplement: S1 Table — (DOCX) [file pgen.1008188.s014.docx]

| **SPECIES** | **SEQUENCE IDENTITY (%)** |
| --- | --- |
| *Dictyostelium fasciculatum* | 28.06 |
| *Mus musculus* | 25.95 |
| *Homo sapiens* | 23 |
| *Arabidopsis thaliana* | 21.21 |
| *Saccharomyces cerevisiae* | 18.7 |
