# Supplementary material for: A telomerase with novel non-canonical roles: TERT controls cellular aggregation and tissue size in Dictyostelium
Source: PLoS Genet. 2019 Jun 25;15(6):e1008188. doi: 10.1371/journal.pgen.1008188 (PMC6592521; doi:10.1371/journal.pgen.1008188)
Supplement: S3 Table — (DOCX) [file pgen.1008188.s016.docx]

| **PRIMER NAME** | **SEQUENCE** |
| --- | --- |
| tert KO 5'arm FP | TAGGTACCAACTTTAACTGACAAG |
| tert KO 5'arm RP | CAAAGCTTAAGGAAATCTCTGTAGT |
| tert KO 3'arm FP | TACTGCAGATTTCATTGTTGGGT |
| tert KO 3'arm RP | AGCGGATCCTTGAATATAAGAAGTT |
| KO confirmation P1 FP | TAGGTTGCCAATGGAGAGAG |
| KO confirmation P2 RP | TTTCTTTTCAGTGTTGTCTGTTG |
| KO confirmation P3 RP | CCAACTTCAATTCTATCTTCATTAC |
| BSR FP | CATTTCGGCAGTACATATTGAAGCG |
