# Supplementary material for: A telomerase with novel non-canonical roles: TERT controls cellular aggregation and tissue size in Dictyostelium
Source: PLoS Genet. 2019 Jun 25;15(6):e1008188. doi: 10.1371/journal.pgen.1008188 (PMC6592521; doi:10.1371/journal.pgen.1008188)
Supplement: S4 Table — (DOCX) [file pgen.1008188.s017.docx]

| **PRIMER NAME** | **SEQUENCE** |
| --- | --- |
| Tert OE FP | CGGAAGCTTAAAAAAATGAATATTCAAAACATTTATAAAG |
| Tert OE RP | GGCGGTACCATAAATTATATTTAAAATATCAATTG |
| rnlA FP_RT | TCCAAGAGGAAGAGGAGAACTGC |
| rnlA RP_RT | TGGGGAGGTCGTTACACCATTC |
