# Supplementary material for: A telomerase with novel non-canonical roles: TERT controls cellular aggregation and tissue size in Dictyostelium
Source: PLoS Genet. 2019 Jun 25;15(6):e1008188. doi: 10.1371/journal.pgen.1008188 (PMC6592521; doi:10.1371/journal.pgen.1008188)
Supplement: S5 Table — (DOCX) [file pgen.1008188.s018.docx]

| **PRIMER NAME** | **SEQUENCE** |
| --- | --- |
| tert FP | ACAACAGACAACACTGAAAAG |
| tert RP | CAAAATGTCTTTCTGAAATTC |
| countin FP | CAACCGGTAATGCTTTTGGT |
| countin RP | CACAAACGAGAGCTGACA |
| smlA FP | TGGATTACACCATGTTCAGCA |
| smlA RP | CCGACTGAAACTGATGCTTTGG |
| acaA FP | CATTCTAGAGGCGGTATTGGC |
| acaA RP | GGAGAAAATGTCTGATTTCGCTT |
| carA FP | ATGTTGGGTTGTATGGCAGTG |
| carA RP | AGGGAAACCACCATTGACAG |
| pdsA FP | CCATTGGGTACAACTGGTGGA |
| pdsA RP | AACTGCCCATGATGGATAGGT |
| regA FP | TAAAGCAACGTTGGCACAAG |
| regA RP | ATGGTGATTCCATTGCTTCC |
| pde4 FP | GATCTTGATACACCAATCGAA |
| pde4 RP | CTTCTGCATCATCTGTACATG |
| 5’nt FP | CAGCTGAACAAGTAGCAATGG |
| 5’nt RP | TGGTGGAAGACTTGATGCTG |
| cadA FP | TTCCAAGAATTGGCTCAAGG |
| cadA RP | CATCAACTGCCCATTGAAAA |
| csaA FP | GCCAAATACAATCGCTGGTG |
| csaA RP | TGGTTGGTGTGAGATCAAAAGC |
| rnlA FP_qRT | TTACATTTATTAGACCCGAAACCAAGCG |
| rnlA RP_qRT | TTCCCTTTAGACCTATGGACCTTAGCG |
